# MiR-30b-5p regulates the lipid metabolism by targeting PPARGC1A in Huh-7 cell line

**DOI:** 10.1186/s12944-020-01261-3

**Published:** 2020-04-16

**Authors:** Qing Zhang, Xue-Feng Ma, Meng-Zhen Dong, Jie Tan, Jie Zhang, Li-Kun Zhuang, Shou-Sheng Liu, Yong-Ning Xin

**Affiliations:** 1 Department of Infectious Disease, Qingdao Municipal Hospital, Qingdao University, Qingdao, 266011 China; 2 Weifang Medical University, Weifang, 261053 China; 3 Hepatology Laboratory, Qingdao Municipal Hospital, Qingdao University, Qingdao, 266011 China; 4 Digestive Disease Key Laboratory of Qingdao, Qingdao, 266071 China; 5 Central Laboratories, Qingdao Municipal Hospital, Qingdao University, Qingdao, 266071 China

**Keywords:** miR-30b-5p, Lipid metabolism, PPARGC1A, NAFLD

## Abstract

**Background:**

MiRNAs are a group of multifunctional non-coding RNAs which play an important role in the various physiological processes including the development of NAFLD. Recent studies have shown that miR-30b-5p tightly associated with the abnormal lipid metabolism in patients with NAFLD, but the detailed mechanism of miR-30b-5p in the lipid metabolism was remain unclear. The aim of this study was to investigate the effect of miR-30b-5p on the lipid metabolism in hepatocellular carcinoma Huh-7 cells.

**Material and methods:**

The correlation of intracellular fat content with the expression of miR-30b-5p in Huh-7 cells and HepG2 cells was investigated by treated cells with different concentrations of FFAs. The effect of miR-30b-5p on the lipid deposition in Huh-7 cells was tested by oil red O staining and TG concentrations measurement. qRT-PCR and western blot were used to investigate the lipid metabolism-related genes PPAR-α, SREBP-1, and GULT1 in miR-30b-5p overexpressed or inhibited Huh-7 cells. Target genes of miR-30b-5p were predicted using starBase, miRDB, and TargetScan databases and verified by qRT-PCR and western blot.

**Results:**

The expression of miR-30b-5p was significant decreased in the FFAs treated Huh-7 cells and HepG2 cells. Overexpressing miR-30b-5p in Huh-7 cells decreased the number and size of lipid droplets and intracellular TG concentrations in Huh-7 cells. Expression of fatty acid oxidation related gene PPAR-α was increased and expression of lipid synthesis related gene SREBP-1 was decreased in the miR-30b-5p overexpressed Huh-7 cells. In addition, miR-30b-5p regulates the intracellular lipid metabolism by targeting PPARGC1A.

**Conclusions:**

Overexpression of miR-30b-5p could reduce the intracellular fat deposition in Huh-7 cells, and miR-30b-5p might regulate the intracellular lipid metabolism by targeting the PPARGC1A in Huh-7 cells.

## Introduction

Non-alcoholic fatty liver disease (NAFLD) is caused by the excessive lipid accumulation in the liver and is thought to be the manifestation of metabolic syndrome in the liver [1]. NAFLD is a complex multifactorial disease involving sedentariness, obesity, poor dietary habit, sarcopenia, insulin resistance, genetic susceptibility, intestinal flora and other factors [2–5]. In addition, NAFLD is closely related to many diseases such as diabetes, cardiovascular disease and kidney disease [6–8]. NAFLD is a multistage liver lesion syndrome that can range from nonalcoholic fatty liver (NAFL) to nonalcoholic steatohepatitis (NASH), liver fibrosis, cirrhosis, and eventual to hepatocellular carcinoma (HCC) [9]. The average prevalence of NAFLD in the world approximate 25%, a latest meta-analysis shows that the overall prevalence of NAFLD in Asia is 29.62% [10]. NAFLD has become one of the most common causes of liver transplantation [11, 12]. With the progress of urbanization, changes in lifestyle and dietary habits, the incidence of NAFLD in China is increasing [13]. Liver biopsy is still the gold standard for the diagnosis of NAFLD, but due to the defect of invasiveness and sampling error, the application of liver biopsy in clinical diagnosis is limited. New noninvasive biomarkers should be exploited to improve the accuracy of NAFLD clinical diagnosis and develop the new therapeutic targets for the treatment of NAFLD [14].

MicroRNAs (miRNAs) are a group of non-coding RNA molecules that about 22 nucleotides in length. MiRNAs mostly interact with the 3′ untranslated region (3’UTR) of their target mRNA molecules to regulate protein synthesis and affect multiple signaling pathways [15]. Mostly, the intracellular miRNAs can exert their functions by binding to the 3′ UTR of target mRNAs directly, some miRNAs can be transferred into nuclear to regulate the expression of target genes. Besides, some miRNAs can be packed into the vesicles and secreted into the extracellular circulation. Extracellular vesicles can be absorbed by other cells through receptor-mediated or receptor-independent ways and release the miRNAs to regulate the gene expressions in the cells [16, 17]. MiRNAs play an important role in tumor development and metabolic diseases, and they possess significant potential and application value in early disease detection, treatment, and monitoring disease progression, as well as organism’s response to various treatments [18].

Previous reports had shown that many miRNAs are closely related to NAFLD, including miR-122, miR-34a, miR-21 and so on [19–21]. In patients with NAFLD, miR-122 was associated with the fibrosis degree, and intrahepatic miR-122 in NAFLD patients was decreased but increased expression of miR-122 was observed in serum [19, 22]. MiR-34a may be involved in the pathogenesis of NAFLD and regulating the plasma lipoprotein metabolism by targeting the HNF4α pathway. Besides, miR-34a/SIRT1/p53 signaling plays a role in the severity of NAFLD [20, 23]. MiRNA-21 partially promotes hepatic lipid accumulation and cancer progression by interacting with the Hbp1-p53-Srebp1c pathway, miRNA-21 also regulates triglyceride and cholesterol metabolism by targeting HMGCR in NAFLD [24, 25]. Recent studies have found that the miR-30 family play an important role in adipocyte differentiation and fat synthesis [26]. A Spanish study conducted by Latorre et al. showed that patients with NAFLD and obesity had lower miR-30b-5p level in the liver compared with simple obese patients, and the expression of miR-30b-5p was decreased in hepatocytes after stimulated with fatty acid in vitro [27]. Dai et al. conducted a study in 2019 showed that higher level of serum miR-30b is correlated with the higher steatosis and HOMA-IR grade [28]. It’s obviously that miR-30b-5p possesses the importance in NAFLD, but the detailed mechanism of miR-30b-5p in the NAFLD is remains unclear. So, we conducted this study to investigate the role of miR-30b-5p in the lipid metabolism in hepatocellular carcinoma cell line (Huh-7). We found that miR-30b-5p could decrease the intracellular triglyceride (TG) level, and regulate the expression of lipid metabolism-related genes Peroxisome proliferator-activated receptor (PPAR-α), Sterol-binding element regulatory protein 1 (SREBP-1), and Glucose transporter 1 (GLUT1). Besides, miR-30b-5p targets the Peroxisome proliferator-activated receptor gamma coactivator 1-alpha (PPARGC1A) to regulate the lipid metabolism in Huh-7 cells.

## Materials and methods

### Cell culture

Huh-7 cell line was purchased from Shanghai Genechem (Shanghai, China) and was derived from the JCRB cell bank which is affiliated with the National Institute for Biomedical Innovation (NIBIO). Huh-7 cells were cultured in DMEM/F-12 medium containing 10% heat-inactivated fetal bovine serum (FBS) (Biological Industries, Israel), 100 units/ml penicillin, 100 μg/ml streptomycin, and incubated at 37 °C incubator with 5% CO_2_ in a humidified atmosphere. When the cell density reached to 90%, the culture medium was removed from the cell monolayer and washed with PBS for twice. After then, 1 ml 0.25% trypsin was added into the cell monolayer to digest 3–4 min. 3 ml complete medium were added into cells to terminate digest and suspended the cells. The cells suspension was divided into 1:3 equally in culture flask and added 4 ml new complete medium into each of the flask.

### Free fatty acids treatment and qRT-PCR

Fat-overloading induction of Huh-7 cell line and HepG2 cell line were conducted as the previous description by Gomez-Lechon and Ricchi [29, 30]. Briefly, Huh-7 cells or HepG2 cells were grown in 6-well cell culture plates. When the cell density reached about 80%, cells were treated with different concentrations (0 mM, 0.5 mM, 1.0 mM) of free fatty acids (FFAs) (The molar mass ratio was palmitic acid (PA): oleic acid (OA) = 1:2). PA and OA were diluted with 1% fatty acid-free BSA. After 24 h treatment with FFAs, cells were collected and the total RNAs of cells were extracted with RNAiso plus (TaKaRa) according to the standard manufacturer’s instructions. Reverse transcription was conducted with the miRcute Plus miRNA First-Strand cDNA kit (TIANGEN, Beijing, China). Quantitative real time polymerase chain reaction (qRT-PCR) was performed using the miRcute enhanced miRNA fluorescence quantification kit (TIANGEN, Beijing, China), and U6 was used as an internal reference to quantify the expression of miR-30b-5p. Primer Informations of miR-30b-5p and U6 were listed in the Table 1.

**Table 1:** Primer sequence for qRT-PCR in this study

Gene	Primer/ Sequences of Product ID of Primer
hsa-miR-30b-5p	F: CD201–0037 (TIANGEN, Beijing, China)
	R: R7015 (TIANGEN, Beijing, China)
U6	F: CD201–0145 (TIANGEN, Beijing, China)
	R: R7015 (TIANGEN, Beijing, China)
PPARGC1A	F: 5′-CCTGTGATGCTTTTGCTGCTCTTG-3′
	R: 5′-AAACTATCAAAATCCAGAGAGTCA-3′
*β*-actin	F: 5′-TGGACTTCGAGCAAGAGATG-3′
	R: 5′-GAAGGAAGGCTGGAAGAGTG-3′

F, forward; R, reverse; PPARGC1A, peroxisome proliferator-activated receptor gamma coactivator 1 alpha

### Lentivirus infection and monoclonal cell lines establishment

The lentivirus of miR-30b-5p overexpression and inhibition were purchased from GeneChem (Shanghai, China). According to the previous reports, the expression of miR-30b-5p can be changed by the infection of overexpressed of inhibited lentivirus [31, 32]. For lentivirus infection, Huh-7 cells were inoculated into a six-well cell culture plate with the density of 1.4 × 10^5^ cells per well. After 24 h incubation, the medium was discarded and then the new medium with a virus titer of MOI 5 were added into each well. After 12 h infection, the virus suspension was replaced with fresh complete medium. After 72 h infection of virus, the fluorescence signals of cell in each well were checked with the fluorescence convert microscope (OLYMPUS IX71, Japan). Cells with positive fluorescence signals were screened with selective medium which was complete medium containing 2 μg/ml puromycin. After 3 days screen, cells that without infection were died completely and the living cells were transferred individually into each well of the 96-well plate and cultured with selective medium containing 1 μg/ml puromycin for 1 month. Total RNAs of each monoclonal cell lines were extracted, reverse transcription and qRT-PCR were performed to test the expression of miR-30b-5p in each of the monoclonal cell lines as above description.

### Oil red O staining

MiR-30b-5p overexpressed Huh-7 monoclonal cells were seeded into 12-well cell culture plate with the density of 3 × 10^5^ cells per well. After cultured for 24 h in serum-free medium, the medium was replaced with 1 ml of complete medium containing 0.5 mM of FFAs. After 24 h incubation, the cell monolayers were stained using oil red O staining kit (Solarbio, Beijing, China). Briefly, the medium of each well were removed and the cell monolayers were washed with PBS for twice. The cell monolayers were fixed with ORO Fixative for 25 min, and washed with distilled water for twice. The cells were immersed with 60% isopropanol for 5 min, discarded the isopropanol and then fresh ORO staining solution were added to stain the cell monolayer for 15 min. Discarded the staining solution and washed the cells with PBS for 5 times. Counterstain was performed by added the Mayer hematoxylin staining solution into each well to stain the nucleus for 2 min, then the cell monolayers were washed with PBS for 5 times. Added the ORO buffer into the wells and incubated for 1 min, then discarded the ORO buffer. Added 1 ml PBS into each well and the cell monolayers were observed and taken photo under the microscope (OLYMPUS BX53, Japan). The area of lipid droplets was counted using the ImageJ software (National Institutes of Health, Bethesda, MD, USA). In detailed, open the Image J software, clicked “File” and selected the cells photo, then clicked “image” and selected “adjust”, next selected “Color Threshold”. In this Interface, adjust saturation and brightness of the photo to turn the area of the stained fat droplets. At the same time, clicked “Original” and “Filtered” to compare the original image with the changed image and checked whether the areas of fat droplets were same. Next, clicked “Analyze” and select “Measure” to measure the area of fat droplets. In consideration of the difference of cell number in different pictures, the cell numbers in each picture were counted. The average lipid accumulation in each picture was equal to area/cell numbers.

### Detection of intracellular TG content

MiR-30b-5p overexpressed and inhibited Huh-7 monoclonal cells were seeded into 12-well cell culture plate with the density of 3 × 10^5^ cells per well. After cultured for 24 h in serum-free medium, the medium was replaced with 1 ml of complete medium containing 0.5 mM FFAs. After 24 h incubation, the content of intracellular TG was detected by triglyceride assay kit (Nanjing Jiancheng Bioengineering Institute, Nanjing, China) according to the manufacturer’s instructions. The absorbance of each sample at 510 nm was read by a microplate reader to calculate the intracellular TG content of each group. The protein concentration of each sample was determined by BCA method to normalize triglyceride content.

### Western blot

MiR-30b-5p overexpressed and inhibited Huh-7 monoclonal cells were cultured in the 12-well plate with DMEM/F-12 complete medium. When then cell density reached 80%, the cells were washed 3 times with PBS and then lysed on ice for 30 min with lysis buffer. The lysate was centrifuged at 12000×g for 10 min to collect the supernatant. The concentrations of protein were determined by the Bicinchoninic acid (BCA) method. Subsequently, 30 μg of each sample was subjected to the 12% sodium dodecyl sulfate-polyacrylamide (SDS-PAGE) gels for electrophoresis. After the electrophoresis complete, the proteins were transferred onto the polyvinylidene difluoride (PVDF) membranes. The membranes were blocked with 5% BSA at room temperature for 1 h prior to incubation with primary antibodies, which included anti-PPAR-α (Species: rabbit; Company: proteintech; Catalog: 15540–1-AP) (1:1000), anti-SREBP-1 (Species: rabbit; Company: Absin; Catalog: abs131802) (1:500), anti-GLUT1 (Species: rabbit; Company: proteintech; Catalog: 21829–1-AP) (1:1000), anti-GAPDH (Species: rabbit; Company: proteintech; Catalog: 10494–1-AP) (1:1000). The membranes were incubated with the primary antibodies overnight at 4 °C and washed 5 times with PBS containing 0.1% Tween-20 (PBST). The membranes were then incubated with HRP-conjugated secondary antibodies (1:5000) at room temperature for 1 h. Then the membranes were washed 5 times with PBST and detected using the ECL reagent (Millipore, America). The signal density of each band was analyzed using ImageJ software (National Institutes of Health, Bethesda, MD, USA) and normalized to GAPDH.

### Identifying target genes of miR-30b-5p

Target genes of miR-30b-5p were predicted using the starBase and miRDB databases. Genes involved in the pathways of lipid metabolism were screened, and the target sites of miR-30b-5p in the 3’UTR of each target gene were predicted by TargetScan database. According to the score by the database and the function of each gene, we selected the PPARGC1A as the potential target gene of miR-30b-5p and verified by the qRT-PCR and western blot, the primary antibody for PPARGC1A (Species: mouse; Company: proteintech; Catalog: 66369–1-Ig) was used. Primer sequences of PPARGC1A for the qRT-PCR were listed in the Table 1, and the *β*-actin was chosen as the internal reference.

### Statistical analysis

Data were represented as the Mean ± SD from three repetition and the differences were analyzed using the Student-*t* test. Data analysis was conducted using GraphPad Prism version 7 (GraphPad Software, CA) and SPSS 22.0 software. Each experiment was repeated in triplicate, *P* < 0.05 was considered statistically significance, *P* < 0.05 was identified as ^*^, *P* < 0.01 was identified as ^**^, *P* < 0.0001 was identified as ^***^.

## Results

### Expression of miR-30b-5p negative correlated with lipid content in Hun-7 cells and HepG2 cells

**Fig. 1:**
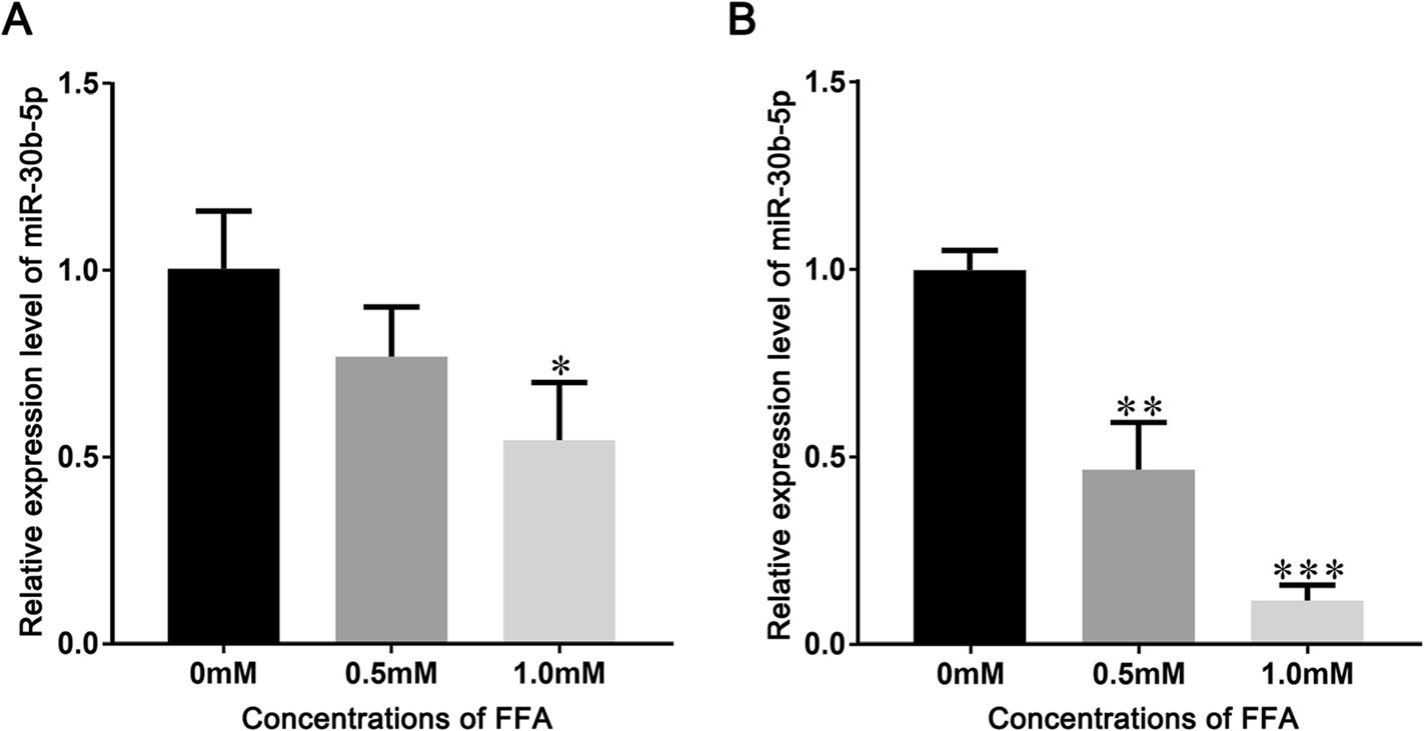
Expression of miR-30b-5p in cells after treated with FFAs. **a** expression of miR-30b-5p in Huh-7 cells after treated with FFAs; **b** expression of miR-30b-5p in HepG2 cells after treated with FFAs

To investigate the effect of lipid overload on the expression of miR-30b-5p in Huh-7 cells and HepG2 cells, we treated the Huh-7 cells and HepG2 cells with 0.5 mM and 1 mM FFAs for 24 h. After FFAs treatment, Huh-7 cells and HepG2 cells were harvested and the expression of miR-30b-5p in Huh-7 and HepG2 were tested by qRT-PCR. As the results shown in the Fig. 1, the expressions of miR-30b-5p in Huh-7 cells and HepG2 cells were significant decreased after the FFAs treatment. These results proved that lipid overload in Huh-7 cells and HepG2 cells could inhibit the expression of miR-30b-5p, suggested that miR-30b-5p may play an important role in the lipid metabolism of Huh-7 cells and HepG2 cells.

### Establishment of miR-30b-5p monoclonal cell strains

**Fig. 2:**
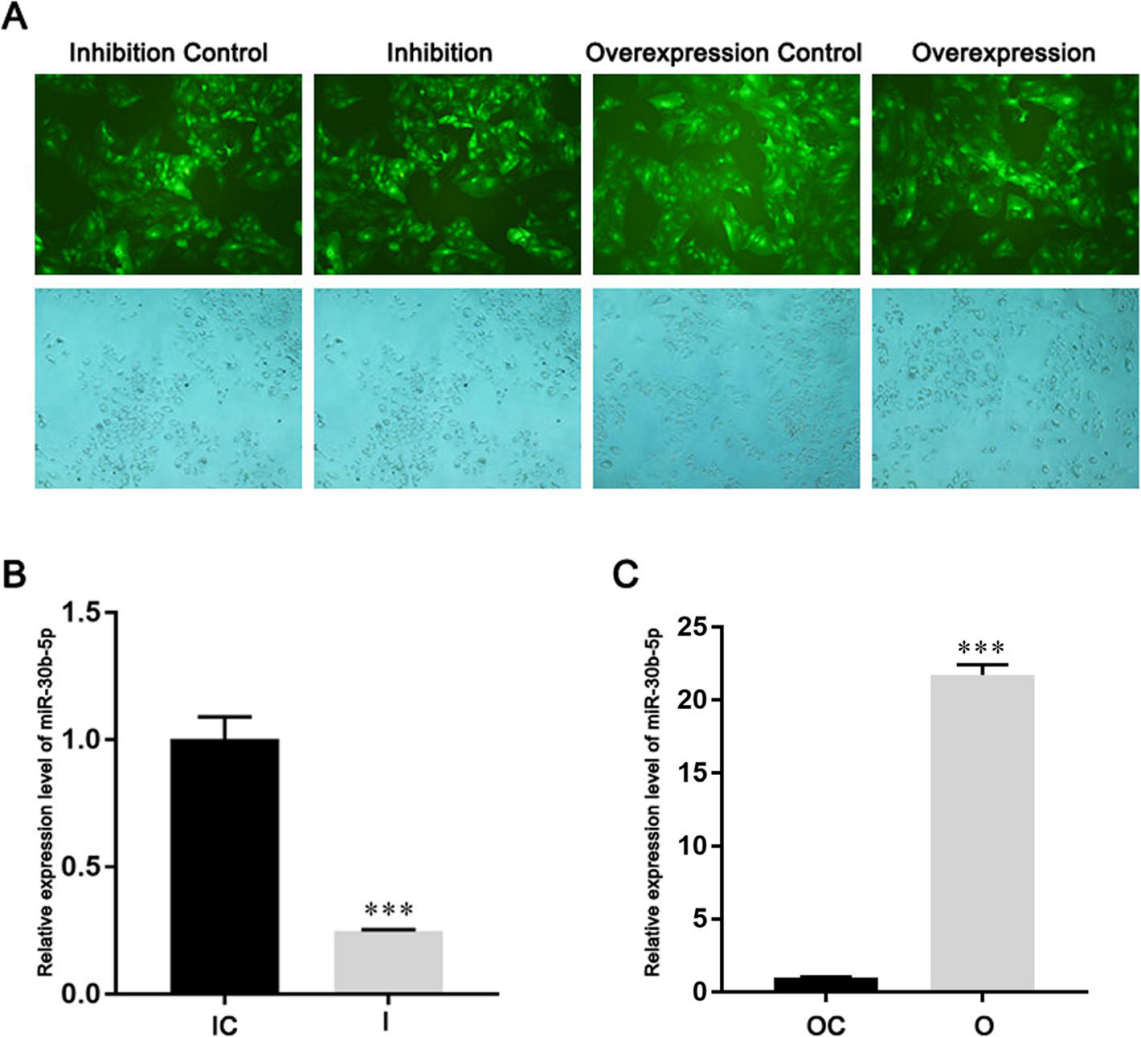
Establishment of miR-30b-5p inhibited or overexpressed monoclonal cell strains. **a** Green fluorescence signal in monoclonal cells; **b** expression of miR-30b-5p in miR-30b-5p inhibited monoclonal cells; **c** expression of miR-30b-5p in miR-30b-5p overexpressed monoclonal cell. (I, inhibition; IC, inhibition control; O, overexpression; OC, overexpression control)

To establish the stable miR-30b-5p overexpressed or inhibited monoclonal cell strains, Huh-7 cells were infected with lentivirus which expresses the miR-30b-5p precursor or inhibitor for 3 days. The positive infected cells were screened by puromycin to establish the stable monoclonal cells. As shown in the Fig. 2a, the green fluorescence could be observed in the miR-30b-5p overexpressed, inhibited and control monoclonal cells, suggested that the lentivirus expressed in the cells successfully. qRT-PCR was used to test the express of miR-30b-5p in the established monoclonal cells. As the results shown, expression of miR-30b-5p was markedly decreased in the miR-30b-5p inhibited monoclonal cells (*P* < 0.001) (Fig. 2b), and the expression of miR-30b-5p were significant increased in the miR-30b-5p overexpressed cells (*P* < 0.001) (Fig. 2c). These results indicated that the miR-30b-5p overexpressed and inhibited monoclonal cells were established successfully.

### MiR-30b-5p regulates lipid accumulation in Huh-7 cells

**Fig. 3:**
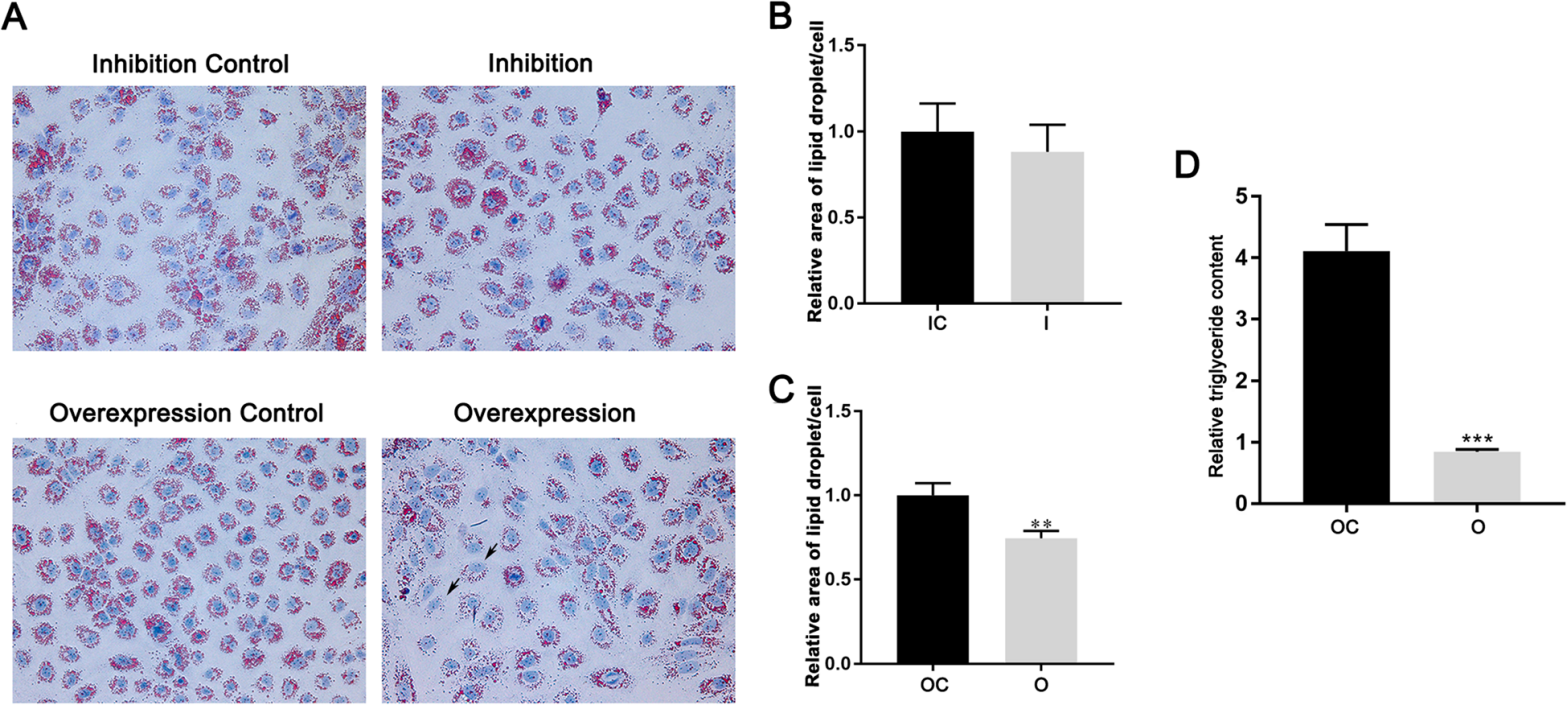
Measurement of intracellular lipid contents and TG concentrations. **a** oil red O staining of the intracellular lipid droplet; **b** relative area of lipid droplet in miR-30b-5p inhibited monoclonal cells; **c** relative area of lipid droplet in miR-30b-5p overexpressed monoclonal cells; **d** relative TG concentrations in the miR-30b-5p overexpressed monoclonal cells and control. (I, inhibition; IC, inhibition control; O, overexpression; OC, overexpression control)

To investigate the role of miR-30b-5p in the lipid metabolism in Huh-7 cells, the monoclonal cells (miR-30b-5p inhibition, inhibition control, miR-30b-5p overexpression, and overexpression control) were treated with 0.5 mM FFAs for 24 h. Then the intracellular lipid contents were determined by oil red O staining and TG concentration measurement. As the results shown in the Fig. 3a and b, there was not obvious difference of the intracellular area of lipid droplet between miR-30b-5p inhibited cells and controls (*P* > 0.05). However, the intracellular area of lipid droplet in miR-30b-5p overexpressed cells was significant decreased compared to the control (*P* < 0.01) (Fig. 3a and c). In addition, we tested the intracellular TG concentrations in each group of the cells. We found that the TG concentrations in the miR-30b-5p overexpressed cells were significant decreased compared to the control (*P* < 0.001) (Fig. 3d), but not statistic difference was observed between miR-30b-5p inhibited cells and control (Data not shown).

### MiR-30b-5p regulates the expression of lipid metabolism-related genes

**Fig. 4:**
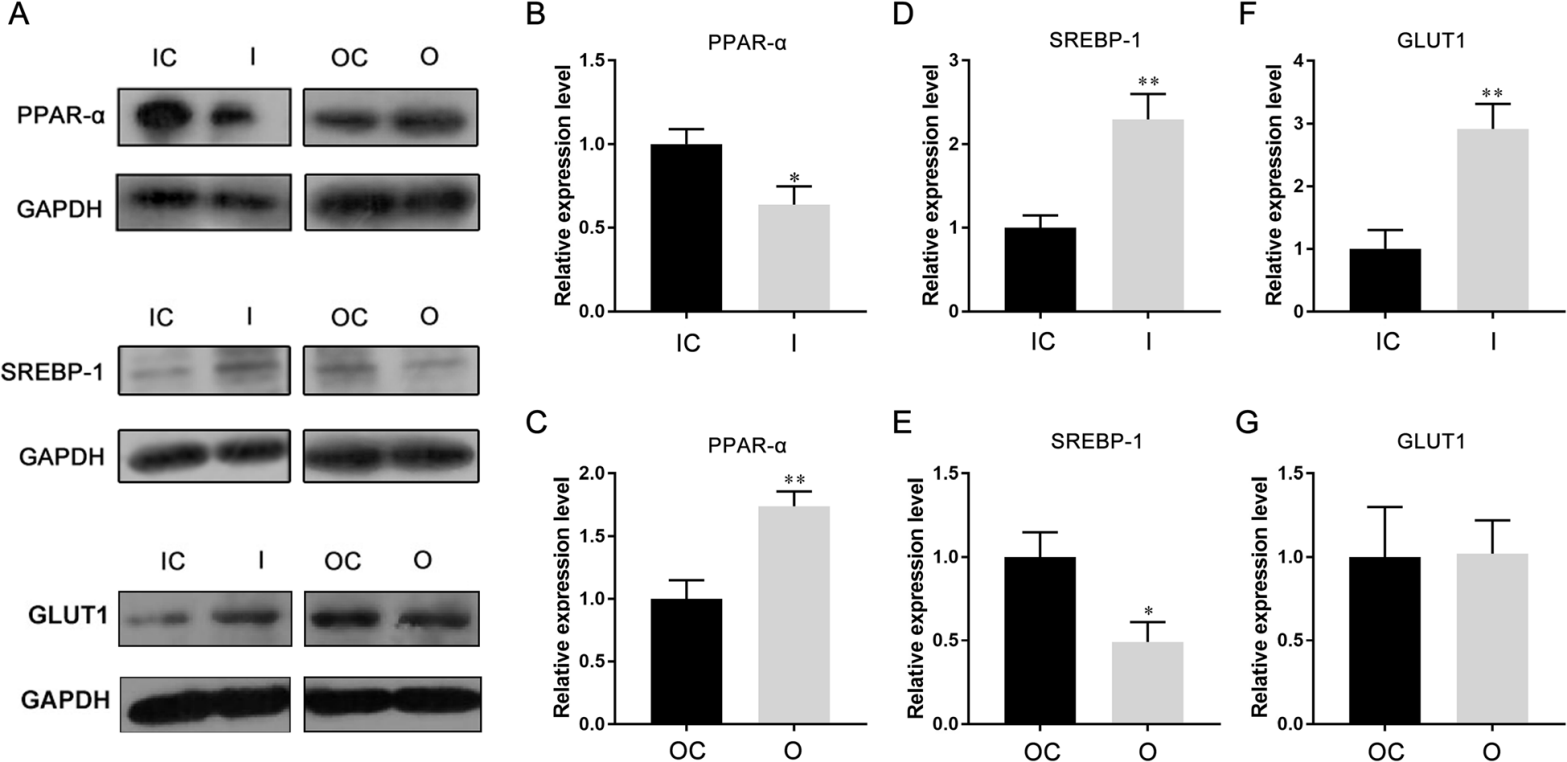
MiR-30b-5p regulates the expression of lipid metabolism-related genes. **a** protein expression of PPAR-α, SREBP-1, and GLUT1 in miR-30b-5p overexpressed or inhibited Huh-7 cells. **b-g** the relative quantify of expression of PPAR-α, SREBP-1, and GLUT1 normalized to GAPDH. (I, inhibition; IC, inhibition control; O, overexpression; OC, overexpression control)

In order to explore the possible pathways of miR-30b-5p participated in the lipid metabolism, we examined the expression of lipid metabolism-related PPAR-α, SREBP-1 and GLUT1 in Huh-7 cells by western blot. As the results shown, inhibition of miR-30b-5p decreased the expression of PPAR-α in Huh-7 cells, and overexpression of miR-30b-5p significantly increased the expression of PPAR-α in Huh-7 cells (Fig. 4a-c). Inhibition of miR-30b-5p significantly increased the expression of SREBP-1 in Huh-7 cells, and overexpression of miR-30b-5p markedly decreased the expression of SREBP-1 in Huh-7 cells (Fig. 4a, d and e). In addition, inhibition of miR-30b-5p significantly increased the expression of GLUT1 in Huh-7 cells, but overexpression of miR-30b-5p did not affect the expression of GLUT1 in Huh-7 cells (Fig. 4a, f and g).

### MiR-30b-5p targets PPARGC1A to regulate lipid metabolism

**Fig. 5:**
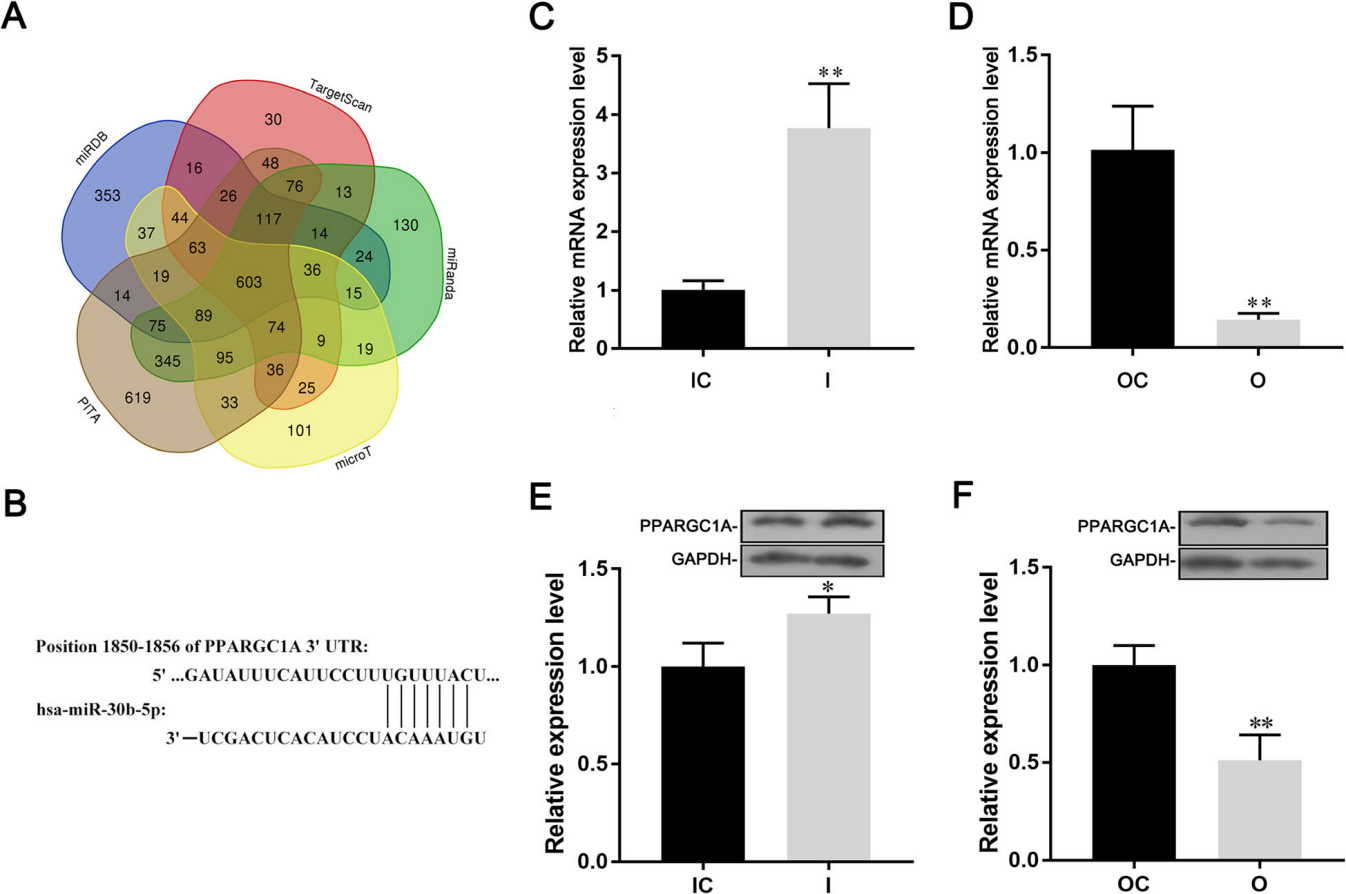
MiR-30b-5p targets PPARGC1A and regulates the expression of PPARGC1A. **a** Predicted target genes of miR-30b-5p by bioinformatics technology; **b** Predicted bind site of miR-30b-5p with the 3’UTR of PPARGC1A; **c** and **d** mRNA expression of PPARGC1A after inhibition or overexpression of miR-30b-5p; **e** and **f** protein expression of PPARGC1A after inhibition or overexpression of miR-30b-5p. (I, inhibition; IC, inhibition control; O, overexpression; OC, overexpression control)

The potential target genes of miR-30b-5p were predicted using the starBase and miRDB databases. We searched miR-30b-5p in starBase database and extracted the predicted target genes in PITA, microT, miRanda, and TargetScan. The results of the PITA, microT, miRanda and TargetScan databases in starBase were used to intersect with the predicted results in miRDB database, ultimately 603 genes which included in both starBase and miRDB databases were obtained (Fig. 5a) [33–35]. GO analysis of these 603 genes was performed by DAVID and KOBAS website to screen the genes involved in lipid metabolism, and 41 lipid metabolism related genes were obtained (Table 2). The bind site of miR-30b-5p and 3’UTR of PPARGC1A was predicted by targetScan website (Fig. 5b). The results of qRT-PCR shown that inhibition of miR-30b-5p could increase the expression of PPARGC1A in Huh-7 cells (Fig. 5c), and overexpression of miR-30b-5p could markedly decrease the expression of PPARGC1A in Huh-7 cells (Fig. 5d). Western blot suggested that the protein expression of PPARGC1A was negative correlated with the expression of miR-30b-5p (Fig. 5e and f).

**Table 2:** Target genes of Hsa-miR-30b-5p that were related to lipid metabolism predicted by starBase and miRDB

Official Symbol	Gene ID	Score	Pathway	Official Symbol	Gene ID	Score	Pathway	Official Symbol	Gene ID	Score	Pathway
ADRA2A	150	98	cGMP-PKG signaling pathway	IRS1	3667	87	Non-alcoholic fatty liver disease (NAFLD)	LYN	4067	87	–
FAM126A	84,668	57	–	CEACAM1	634	57	**–**	**PPARGC1A**	**10,891**	**93**	AMPK signaling pathway
ELOVL5	60,481	88	Fatty acid metabolism	GATA6	2627	60	–	NT5E	4907	99	Metabolic pathways
NR5A2	2494	94	Maturity onset diabetes of the young	PIP4K2B	8396	89	Phosphatidylinositol signaling system	LCLAT1	253,558	99	Glycerolipid metabolism
PDSS1	23,590	92	Biosynthesis of secondary metabolites	CERS6	253,782	82	Sphingolipid signaling pathway	BCL11B	64,919	93	Transcriptional misregulation in cancer
IDH1	3417	90	Citrate cycle (TCA cycle)	PIK3CD	5293	93	AMPK signaling pathway	NUS1	116,150	85	Biosynthesis of secondary metabolites
VAV3	10,451	78	cAMP signaling pathway	PLAGL2	5326	98	–	MYO5A	4644	92	Pathogenic *Escherichia coli* infection
IP6K3	117,283	91	Phosphatidylinositol signaling system	SIRT1	23,411	63	Glucagon signaling pathway	IRS2	8660	74	AMPK signaling pathway
NRBF2	29,982	64	Autophagy - animal	SNAI1	6615	88	Adherens junction	PI4K2B	55,300	85	Metabolic pathways
DGKZ	8525	65	Glycerolipid metabolism	ST8SIA4	7903	87	–	ERLIN1	10,613	94	–
MBOAT1	154,141	86	Glycerolipid metabolism	LARGE1	9215	94	Metabolic pathways	GPCPD1	56,261	83	Glycerophospholipid metabolism
DOLPP1	57,171	97	N-Glycan biosynthesis	CAT	847	52	Carbon metabolism	CYP24A1	1591	98	Metabolic pathways
PER2	8864	93	Circadian rhythm	B3GNT5	84,002	100	Metabolic pathways	PLA2G12A	81,579	81	Fat digestion and absorption
PIGA	5277	81	Metabolic pathways	PIP4K2A	5305	99	Metabolic pathways				

## Discussion

MiRNAs as a group of regulatory non-coding RNAs, play an important role in the physiological process of host [36]. Accumulated studies had concerned the association of miRNAs with the metabolic disorders in NAFLD patients [37]. Previous reports have shown that the expression of miR-30b-5p in NAFLD patients was decreased significantly, but no detailed experiments were conducted to investigate the role of miR-30b-5p in the lipid metabolism. In this study, we investigated the role of miR-30b-5p in lipid metabolism in Huh-7 cells and HepG2 cells. We found that the expression of miR-30b-5p in Huh7 cells and HepG2 cells were markedly decreased after FFAs treatment, which resulting in the cellular lipid accumulation. These results conform to the previous study that the expression of miR-30b-5p was decreased in NAFLD patients compared with NAFLD-free patients [27].

In this study, we focused our attention on the effect of miR-30b-5p in lipid accumulation. Palomer et al. reported that palmitic acid could induce insulin resistance while oleic acid could attenuate inflammation, impaired function of cellular organelles [38]. Besides, Matteo et al. found that in three hepatocytic cell lines (HepG2, Huh-7, WRL68), cells treated with oleic acid had larger steatosis extent but less apoptotic compared with treated with palmitic acid [30]. However, Gómez-Lechón et al. reported that the FFAs mixture containing a low proportion of palmitic acid (oleate/palmitate, 2:1 ratio) was associated with minor toxic and apoptotic effects, thus representing a cellular model of steatosis that mimics benign chronic steatosis [29]. Furthermore, oleate/palmitate in a ratio of 2:1 to establish lipid overload model in cells has been widely adopted [39, 40].

Intracellular abnormal lipid metabolism could lead to the fat deposition especially the accumulation of TG. In this study, we used the oil red O staining to measure the fat deposition. We found that overexpression of miR-30b-5p could significantly decrease the area of lipid droplet in Huh-7 cells. In addition, the concentrations of TG also decreased in the miR-30b-5p overexpressed Huh-7 cells. Expression of miR-30b-5p was inhibited by FFAs in Huh-7 cells, indicated that miR-30b-5p might positively regulates the lipid deposition in cells, when the expression of miR-30b-5p were increase, the intracellular lipid droplet and TG content were decreased markedly. These results suggested that miR-30b-5p could regulate lipid metabolism in Huh-7 cells. Qin et al. reported that miR-30b-5p was down-regulated in HCC tissues and hepatocyte cell lines, and miR-30b-5p could repressed the cell proliferation and cell cycle of HCC cells [41]. Nevertheless, this role of miR-30b-5p did not affect our study. In our study, we mainly investigated the role of miR-30b-5p in the lipid metabolism in Huh-7 cells. The quantitative experiments were conducted to adjust the differences of numbers of cells using western blot, qRT-PCR, or cell counting.

PPAR-α is a key regulator of lipid metabolism, which could promote the fatty acid oxidation in cells [42]. SREBP-1 is a transcriptional activator of lipid metabolism that could promote the lipid synthesis [43]. GLUT1 is a glucose transporter that is responsible for constitutive or basal glucose uptake [44]. In order to investigate the detailed mechanism of miR-30b-5p in the lipid metabolism, we tested the lipid metabolism-related genes PPAR-α, SREBP-1, and GLUT1, which were involved in the fatty acid oxidation, lipid synthesis, and glucose uptake. According to the results, when the miR-30b-5p was overexpressed in the Huh-7 cells, the fatty acid oxidation was accelerated and the lipid synthesis was inhibited, but the glucose uptake did not changed, as reflected by the higher expression of PPAR-α, the lower expression of SREBP-1, and the unchanged expression of GLUT1. When the miR-30b-5p was inhibited in the Huh-7 cells, the fatty acid oxidation was inhibited, the lipid synthesis glucose uptake was accelerated, as reflected by the higher expression of SREBP-1 and GLUT1, and the lower expression of PPAR-α.

We predicted the potential target genes of miR-30b-5p using the starBase and miRDB databases, and one of the possible target genes was PPARGC1A. MiR-30b-5p could regulate the mRNA and protein expression of PPARGC1A in Huh-7 cells. PPARGC1A plays an important role in NAFLD and involves mitochondrial oxidative phosphorylation, gluconeogenesis and fatty acid synthesis [45]. In 2004, Koo et al. reported that increased hepatic insulin sensitivity was occurred in PPARGC1A-deficient mice [46]. Park et al. found that expression of PPARGC1A was increased in the liver of high-fat diet mice, and expression of PPARGC1A increased in AML12 and H4IIE cells after palmitic acid stimulation [47]. In Huh-7 cells, palmitate could up-regulate the expression of PPARγ by targeting PPARGC1A, and palmitate could up-regulate PPARGC1A expression in the liver of NAFLD mice [48]. All these reports supported our conclusion that PPARGC1A possesses the significant role in the regulation of lipid metabolism, but the role of PPARGC1A in NAFLD remains controversial. A review by Piccininë et al. in March 2019 reported that the expression of PPARGC1A was decreased in NAFLD, and the expression of PPARGC1A was negatively correlated with the severity of NAFLD [49]. To clarify the correlation between PPARGC1A and NAFLD, further studies are needed.

## Conclusion

In summary, our study proved that expression of miR-30b-5p could be inhibited by the FFAs in Huh-7 cells and HepG2 cells. Overexpression of miR-30b-5p could inhibit the fat deposition and decrease the content of TG in Huh-7 cells. MiR-30b-5p could regulate the expression of lipid metabolism-related genes such as PPAR-α, SREBP-1, and GLUT1. In addition, our results indicated that miR-30b-5p participates in the lipid metabolism by targeting the PPARGC1A genes. Our study suggested that miR-30b-5p plays an important role in the lipid metabolism and development of NAFLD.

## Data Availability

The datasets used and/or analyzed during the current study are available from the corresponding author on reasonable request.

## Abbreviations

FBS: Fetal bovine serum
FFA: Free fatty acid
GLUT1: Glucose transporter 1
HCC: Hepatocellular carcinoma
MiRNAs: MicroRNAs
NAFL: Nonalcoholic fatty liver
NAFLD: Non-alcoholic fatty liver disease
NASH: Nonalcoholic steatohepatitis
PPAR-α: Peroxisome proliferator-activated receptor
PPARGC1A: Peroxisome proliferator-activated receptor gamma coactivator 1-alpha
qRT-PCR: Quantitative real time polymerase chain reaction
SREBP-1: Sterol-binding element regulatory protein 1
TG: Triglyceride
UTR: Untranslated region
